# Trends and Sources of Crime Guns in California: 2010–2021

**DOI:** 10.1007/s11524-023-00741-y

**Published:** 2023-09-11

**Authors:** Hannah S. Laqueur, Christopher McCort, Colette Smirniotis, Sonia Robinson, Garen J. Wintemute

**Affiliations:** 1 Violence Prevention Research Program, Department of Emergency Medicine, University of California, Davis, USA; 2 California Firearm Violence Research Center, Davis, USA

**Keywords:** Crime guns, Gun theft, Ghost guns, Criminal gun markets, Handgun purchasing

## Abstract

Firearm-related interpersonal violence is a leading cause of death and injury in cities across the United States, and understanding the movement of firearms from on-the-books sales to criminal end-user is critical to the formulation of gun violence prevention policy. In this study, we assemble a unique dataset that combines records for over 380,000 crime guns recovered by law enforcement in California (2010–2021), and more than 126,000 guns reported stolen, linked to in-state legal handgun transactions (1996–2021), to describe local and statewide crime gun trends and investigate several potentially important sources of guns to criminals, including privately manufactured firearms (PMFs), theft, and “dirty” dealers. We document a dramatic increase over the decade in firearms recovered shortly after purchase (7% were recovered within a year in 2010, up to 33% in 2021). This corresponds with a substantial rise in handgun purchasing over the decade, suggesting some fraction of newly and legally acquired firearms are likely diverted from the legal market for criminal use. We document the rapid growth of PMFs over the past 2–3 years and find theft plays some, though possibly diminishing, role as a crime gun source. Finally, we find evidence that some retailers contribute disproportionately to the supply of crime guns, though there appear to be fewer problematic dealers now than there were a decade ago. Overall, our study points to temporal shifts in the dynamics of criminal firearms commerce as well as significant city variation in the channels by which criminals acquire crime guns.

**Supplementary Information:**

The online version contains supplementary material available at 10.1007/s11524-023-00741-y.

## Introduction

Firearm-related interpersonal violence is a leading cause of death and injury in cities across the United States. A critical gap in our understanding of the problem is highlighted by our inability to provide a satisfactory answer to the question: where do crime guns come from? While trace data on firearms confiscated by police and submitted to the Bureau of Alcohol, Tobacco, Firearms, and Explosives (ATF) to identify the first retail sale have provided one means of better understanding the illegal supply of firearms to criminals, restrictions on record sharing since adoption of the Tiahrt amendments in the early 2000 s have precluded detailed trace data research, other than at the local level [1, 2]. However, in California, since 2002, the state has required that local law enforcement agencies submit information on all recovered crime guns to its Department of Justice (CADOJ) to be forwarded to ATF for tracing and that the information be retained by CADOJ for at least 10 years and be available for research [3, 4]. The present study is the first to analyze these California data.

In this paper, we provide a descriptive analysis of crime gun reports from 2010 to 2021 and examine several potentially important sources of crime guns, including privately manufactured firearms (PMFs), firearm theft, and licensed retailers who may intentionally or through negligence break the law. We rely on a unique constellation of datasets that include records for over 380,000 crime guns recovered in the state, and more than 126,000 guns reported stolen, linked to individual firearm purchase records dating back to 1996 for handguns and 2014 for long guns. These Dealer Records of Sale (DROS) transactions allow us to follow or “trace” firearms from their first recorded transaction in the state to the one most immediately preceding their use in a crime. This is distinct from ATF trace records, which only record the first legal sale.

We begin by describing trends in crime gun recovery, mapping time-to-crime (TTC)—the time between retail sale and the recovery of the gun by law enforcement at a crime scene or from a criminal suspect—across place and over time. We examine trends in firearms recovered in violent crimes and other offenses. We also describe the rise of privately manufactured firearms (PMFs) in crime gun recoveries. Next, we turn to the relationship between firearm theft and criminal use. We present temporal trends in theft in the state and link firearms reported stolen to legal transaction and crime gun recovery records to trace the “life course” of these weapons. We then investigate the role of retail dealers in supplying firearms to criminals, examining whether there are certain dealers who are more likely to sell crime guns, the characteristics of these dealers, and trends over time.

## Data

The principal data for this study are California gun trace records. These data include specifics about the recovered gun including place of recovery and the crime in which it was used. From 2010 to 2021, the data contain records for 381,213 recovered guns. This represents between 86% and 91% of the total number of annual trace records reported by ATF for the state, depending on year. In all but a handful of jurisdictions, there does not seem to be any significant missingness across years. We estimate inconsistent reporting in the handful of jurisdictions amounts to less than 5% of total crime gun reports over the study period. (Details on our assessment of data reporting consistency and quality across cities are provided in the Supplement Section 1, with cities with suspected under-reporting shown in Supplement Fig. 1).

In addition to the crime gun recovery records, we have records for 126,941 firearms reported stolen from 2010 to 2021. California is one of 15 states that require private citizens to report to law enforcement when a firearm is lost or stolen [5]. Since July 2017, individuals must report within 5 days of the time they discovered or reasonably should have discovered the loss or theft [6].

**Fig. 1:**
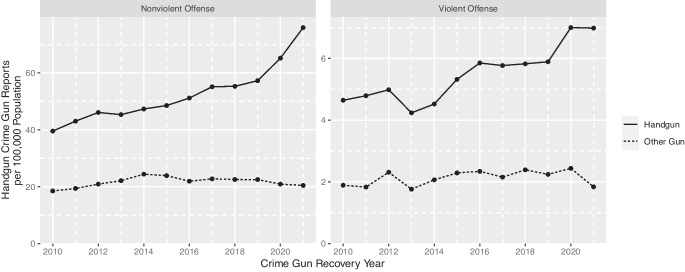
Trends in crime gun recoveries in violent and non-violent crimes: 2010–2021

We linked the crime gun recovery records and stolen gun reports to California’s Dealer Record of Sale (DROS) data, maintained in the CADOJ’s Automated Firearm System (AFS). These records contain all handgun transactions since 1996 and transactions for rifles and shotguns since 2014. California law requires that essentially all transfers of firearms be done through a Federal Firearms Licensees retailer (FFL), including transfers between private parties, gun show sales, gifts, loans, and pawned or consigned weapon redemptions. Prospective firearm purchasers must submit an application to the FFL, who provides purchaser information to CADOJ through electronic transfer. CADOJ then checks state and federal records to determine whether the applicant is legally disqualified from purchasing or possessing firearms under state or federal law. The DROS records include the prospective purchaser information (name, date of birth, sex, race/ethnicity, address); date and time of transaction; the type of transaction (e.g., sale, denial, transfer, pawn); and identifiers for the seller.

We constructed the ownership history of recovered crime guns and stolen guns by linking these records to DROS transactions using the CADOJ linkage number. We linked a total of 202,396 crime guns and stolen guns to DROS transactions. The crime gun data do not consistently include possessor information. Nor are we able to identify purchases that occurred in other states, as ATF traces do. However, unlike ATF trace data, we are able to identify not only the first purchase, but all subsequent legal transactions. This updated measure still does not give us an estimate of how long the gun was in the possession of the offender from whom it was recovered, but, compared to the standard ATF measure, does provide a lower ‘upper bound.’ Given a significant majority of recovered crime guns are handguns (70%), and the DROS data are incomplete for long guns during our study period, we focus our temporal analyses such as trends in TTC on handguns.

## Trends in Crime Gun Recoveries and Time-to-Crime

The number of crime guns recovered in the state has grown over the last decade by close to 70%, from a rate of 64.6 per 100,000 population to 107.2 per 100,000 in 2021. Figure 1 shows the separate trends for handgun recoveries and long gun recoveries, broken down by recoveries in a violent crime and recoveries connected to other offenses (Supplement Table 1 details the CADOJ crime categories included in violent crime). Recoveries of handguns in both violent crimes and other crimes have close to doubled. Notably, long gun recoveries have been constant over the study period, which provides some assurance that the rise in crime gun recoveries is not an artifact of increased law enforcement reporting. Crime gun recoveries have increased for most crime categories, though most significantly for weapon offenses: there were approximately 15,000 weapon offense recoveries in 2010 and close to 30,000 in 2021; violent crime recoveries grew from approximately 1,600 in 2010 to 2,300 in 2021 (Supplement Fig. 1).

Over our study period, there has been a substantial increase in the number of crime guns recovered with a short TTC. Short TTC is a commonly used indicator of illicit trafficking or transfer [7]. Less than 3 years is generally considered an indicator of possible illegal activity by dealers or traffickers; a time of less than a year a very strong indicator [2, 8, 9]. Research has also shown that TTC is longer, on average, in states with more stringent firearm laws and regulations on dealers, suggesting that these laws can reduce the ease with which firearms are illegally diverted from legal commerce [10, 11]. Privately manufactured firearms (PMF) are not included in this calculation as there is no initial retail sales date from which to calculate a time to crime.

**Fig. 2:**
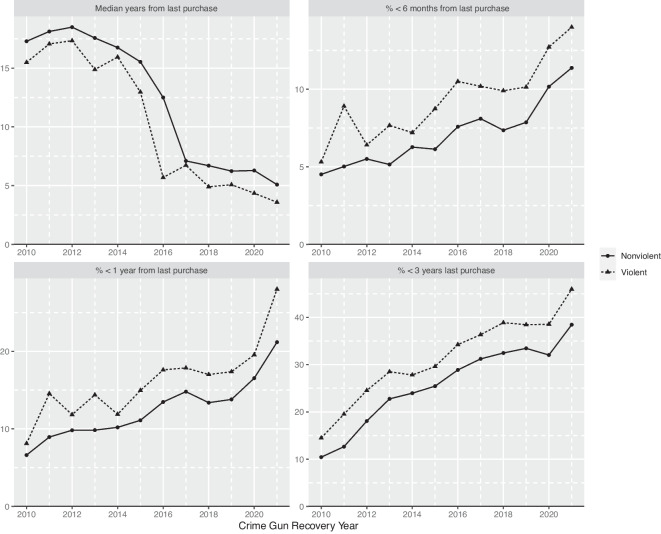
Trends in time-to-crime: 2010–2021

TTC is consistently shorter for handguns recovered in a violent crime compared with other crime gun recoveries (Fig. 2). In all cases, median TTC has declined over the past decade. From 2010 to 2021, the median TTC for handguns recovered in violent crime dropped from 15 years to 4 years, with a particularly steep decline between 2014 and 2018. The percentage of crime guns recovered within a short period (6 months, 1 year, and 3 years) has increased significantly over the decade. For example, for handguns recovered in a violent crime, the fraction recovered within 6 months has nearly tripled, from 5% in 2010 to close to 15% in 2021, with the most significant rise in the last 4 years; the percent recovered within 1 year has more than tripled from 7% to 33%, with a dramatic increase in 2021 from the year prior. These TTC numbers are calculated from the *last* known transaction. As noted previously, ATF statistics report TTC from the first point of sale. Overall, the difference in median TTC during our study period calculated from these two methods is approximately 2 years: 12.8 years using the last known transaction, 14.9 years using the first. Regardless of whether we use first or last purchase, we see a growing number and share of short TTC crime guns.

**Fig. 3:**
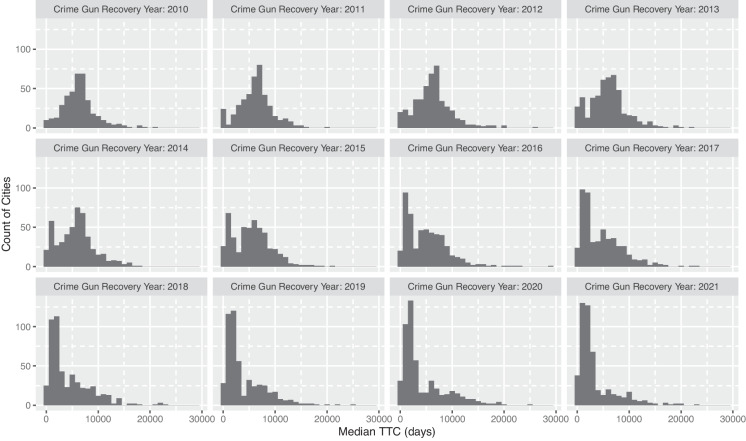
Median TTC across California cities: 2010–2021

We find this same state-level pattern of shorter median TTC and rising short TTC crime across California cities. Figure 3 shows TTC across all cities (that had at least 10 crime gun recoveries per year) 2010–2021. In 2010, the distribution is roughly normally distributed and centered around the median (approximately 15 years), it begins to shift in the twenty-teens, and by the last 4 years of data, most California cities have a median TTC that is less than half what it was in 2010.

This rise in short TTC may in part be the result of the rise in firearm purchasing over the past decade. There were more than three times as many handgun purchases in California in 2020 as compared to 2010 (666,168 vs. 217,836); handgun purchasing was down in 2021 (519,806) but still up by 140% from 2010 (Supplement Fig. 1 shows monthly sales over the study period). The Federal Bureau of Investigation’s (FBI) National Instant Criminal Background Check System (NICS), a commonly used proxy for firearm sales, shows a similar national rise in purchasing over the last decade and spike in purchasing in 2020. In 2010, there were close to 15 million NICS checks; there were over 28 million in 2019 and almost 40 million in 2020 [12].

National ATF trace data also indicate a rise in recovered crime guns with a short TTC in the last few years. The percentage of traces with a TTC less than 1 year (measured from first purchase to time of recovery) was relatively stable between 2017 and 2019, but increased from 20% in 2019 to 32% in 2021 [13]. This national increase in firearm purchasing and rise in short TTC has also been connected to the rise in firearm homicides seen in 2020 and 2021, both in California and across the USA [14, 15]. Nationwide, the firearm homicide rate increased by close to 35% from 2019 to 2020 and by an additional 8% in 2021 [16]. Firearms were used in 81% of the homicides perpetrated in 2020, the highest proportion ever reported [16, 17]. In California, from 2019 to 2021, homicides rose by 41% and aggravated assaults by 18%, driven by a rise in firearm-related homicides and aggravated assaults, which were up 52% and 64%, respectively [18]. There are obviously many means by which potential offenders can obtain firearms, but these trends suggest that some fraction of the newly and legally acquired firearms in the population are likely diverted from the legal market for criminal use.

## The Rise of Ghost Guns

We now turn to the role of criminal access to untraceable PMFs. These so-called “ghost guns” have been growing in popularity across the USA, but have been especially prevalent in states such as California with strict firearm laws [19]. Unserialized, untraceable firearms are built from unfinished parts that do not meet the legal definition of a firearm and thus are not regulated under federal law. California is one of a handful of states that have begun to require such firearms to be registered. Since 2019, owners must obtain a unique serial number from the CADOJ for any PMF [20], and sales of firearm precursor parts, including unfinished receivers, must be conducted through licensed dealers [21].

**Fig. 4:**
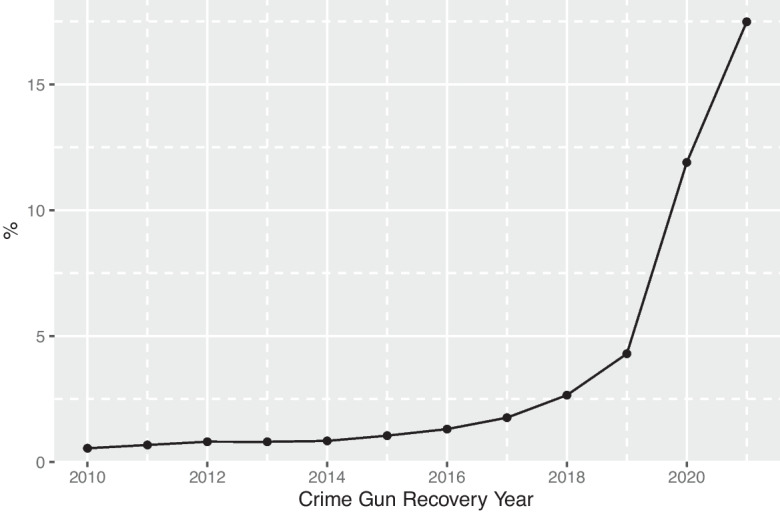
Trends in Ghost Gun Recovery: 2010–2021

To estimate the prevalence of recovered PMFs, we use several proxies including manufacturer code “unknown” and manufacturer code “unknown and manufactured in the USA but not military-issued.” Using these estimates, Fig. 4 shows the introduction of PMFs into the criminal gun market around 2015–2016 and a dramatic increase in their prevalence in the last 2 years, reaching approximately 20% of all recovered crime guns in the last year for which we have data. Some share of this growth may simply be the enforcement of the 2019 law requiring PMF registration with CADOJ. We therefore also look at recoveries for non-weapon offenses and violent offenses separately. This estimate will be conservative as some weapon offenses involve more than simple illegal possession. Statewide, in 2021, 13% of firearms recovered in violent crimes were PMFs and approximately 14% of firearms recovered in non-weapon offenses were PMFs.

**Fig. 5:**
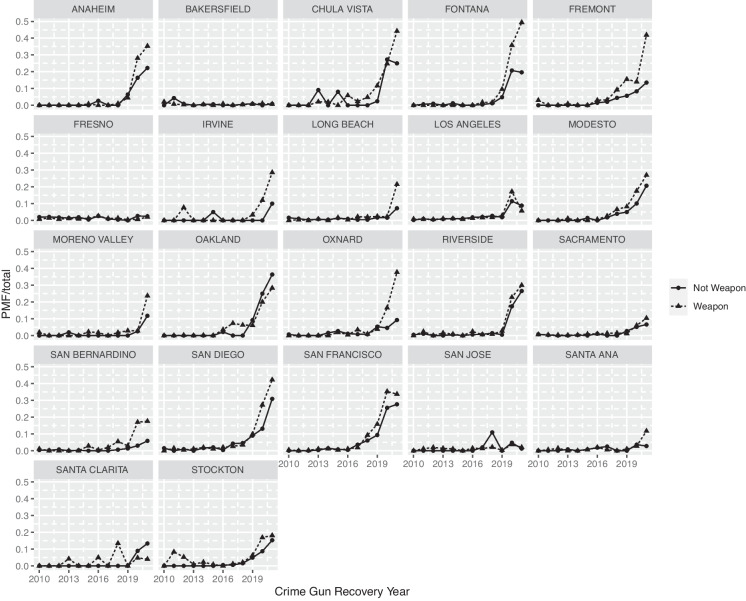
Trends in Ghost Gun Recovery by City: Weapon Offenses vs Other Crimes

Figure 5 shows trends across medium and large California cities (those with population of 200,000 or more) in the proportion of crime guns that were PMFs. We present weapon offense and other crime gun recoveries separately to disambiguate use in crime from the simple possession of an unregistered PMFs. The data show substantial variation in the prevalence of PMFs in the criminal gun market across place. For example, in San Francisco and San Diego, close to 30% of recovered crime guns were PMFs. In Los Angeles, the prevalence appears to be much less, reaching only 10% in 2020.

Consistent with the rise in PMF recoveries in crime, the share of recovered crime guns with prior legal transaction records in California has declined significantly in the last few years. From 2010 to 2019, each year approximately 40–43% of recovered handguns had at least one transaction record in DROS; the percentage dropped to 33% in 2020 and 28% in 2021 (Supplement Fig. 1).

However, this decline in the share of crime guns with prior sales records in California does not appear to be solely related to PMFs: aggregate ATF trace data also indicate an increasing share of recovered firearms sourced from other states (Supplement Fig. 1). In 2010, ATF reported that 15% of California recovered crime guns were first purchased in another state; by 2021, 29% originated in a different state [22]. Studies have consistently found that states with stricter gun laws such as California have a higher proportion of crime guns originating from other states compared to states with lax gun laws [8, 11, 23]. Likewise, studies have documented the earlier and more dramatic growth in the prevalence of PMFs in states with strict gun laws, likely reflecting the demand among prohibited persons for firearms via alternative channels [19].

## Stolen Guns and Crime Gun Recoveries

We now turn to examining the role of theft in diverting firearms from the legal market and supplying firearms to offenders. The extent to which theft is an important source of firearms to criminals remains debated [24]. The Bureau of Justice Statistics (BJS) National Survey of Prison Inmates data suggests that theft plays a relatively minor role in directly arming criminals. The most recent 2016 survey found that among prisoners who reported that they possessed a firearm during their offense, 6% said that they had stolen the firearm [25]. The most common source reported was the illicit market (43%); 25% said they had obtained the firearm from a family member or friend or as a gift, 7% reported that they found it at the scene of the crime, and 7% reported that they had purchased it under their own name from a licensed firearm dealer. Of course, even if the possessor did not directly steal the weapon, theft could source firearms to offenders if there are traffickers who steal firearms and sell them on the illicit market.

Overall, during our study period, approximately 8% of recovered crime guns had previously been reported stolen. This fraction is somewhat higher than other estimates from single jurisdictions [24]. This is likely because we have access to data across the state, whereas local law enforcement agencies do not check thefts in other cities. Even with statewide data, to the extent that firearms are stolen but not reported to law enforcement, estimates of the fraction of crime guns that had been stolen will be a lower bound.

**Fig. 6:**
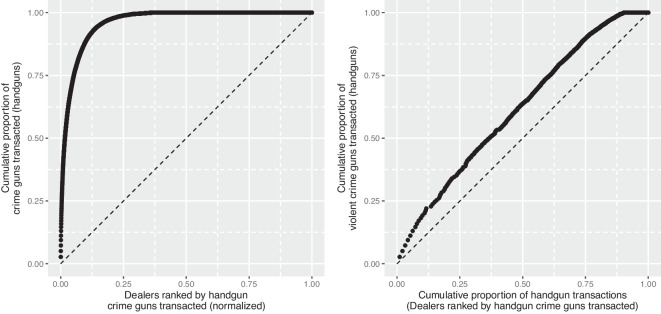
Dealer Distribution of Sales and Crime Guns

In recent years, both the number of stolen gun reports, as well as the share of recovered guns previously reported stolen, have fallen. The rate of stolen gun reports reached a high of 3.3 per 100,000 in 2012–2013 (close to 12,000 stolen firearms per year); it was down to less than 1.5 in 2020 and 2021 (a total of 7,906 stolen firearm reports in 2021) (Supplement Fig. 1). The fraction of recovered crime guns previously reported stolen has also dropped over the last few years: between 2010 and 2018, approximately 6–7% of firearms recovered in a violent crime had been reported stolen; in 2021, just under 4% of firearms recovered in a violent crime had been reported stolen (Supplement Fig. 1). Insofar as California’s 2017 state law requiring owners to report lost and stolen weapons within 5 days [26] has had any impact on reporting, the number of thefts may have dropped even more than the data reflect.

This decline in both the number of stolen guns and share of stolen crime guns is consistent with the hypothesis that theft is playing a diminishing role in arming criminals as individuals seeking firearms for illegal use are increasingly able and likely to acquire PMFs. The decline is also consistent with the more general state trend in declining theft, particularly in the last 2 years during the COVID-19 pandemic and related lock-downs. The 2020, statewide property crime rate was 9% lower than it was in 2019, and the lowest observed since 1960 [18]. Larceny was down 15%, an offense that, like robbery, tends to increase with the volume of social interactions; burglary was down 4%, with more people at home and fewer houses unattended [27]. On the other hand, auto theft increased by 20% in 2020 compared to the year prior, [27] likely related to more vehicles being left unattended. Nationally, gun theft from vehicles has risen over the past decade, with some research suggesting that this may be linked to increasingly permissive firearm carry laws [28].

While statewide trends suggest some reduction in stolen firearms, there is substantial variation across jurisdictions in the state (see Supplement Fig. 1). In San Francisco and Sacramento, for example, the percentage of crime guns that were previously reported stolen reaches 15–17% in several years; in Los Angeles and San Diego, the fraction of crime guns previously stolen ranges from 5% to 10% during all years of data. The firearms that are recovered in a city may have been originally stolen and reported in a different city. For example, we find firearms reported stolen in Chula Vista are more likely to be recovered in San Diego; firearms reported stolen in Irvine are more likely to be recovered in Los Angeles (see Supplement Fig. 1). This suggests a flow of stolen firearms from some suburbs to proximal cities.

Finally, we find a significant fraction of firearms reported stolen never appear to be used in crime. Statewide, among firearms reported stolen, the chance of recovery within a year ranges from 5% in 2010 to 8% in 2021 (see Supplement Table 1). Among those that are recovered, recovery occurs fairly soon after the theft: the median time between theft and recovery is less than 1 year (257 days). The cumulative recovery rate over our study period is only 15%. Thus, if a stolen firearm is not recovered within the first few years, it appears to be unlikely that it will ever be recovered. What fraction of the remaining 85% of stolen guns are used in crimes that simply go undetected is unknown. What is clear from our data is that once a firearm is stolen, it does not appear to re-enter *legal* circulation in the state: among stolen guns, only 1.3% had subsequent records of transaction in DROS.

**Fig. 7:**
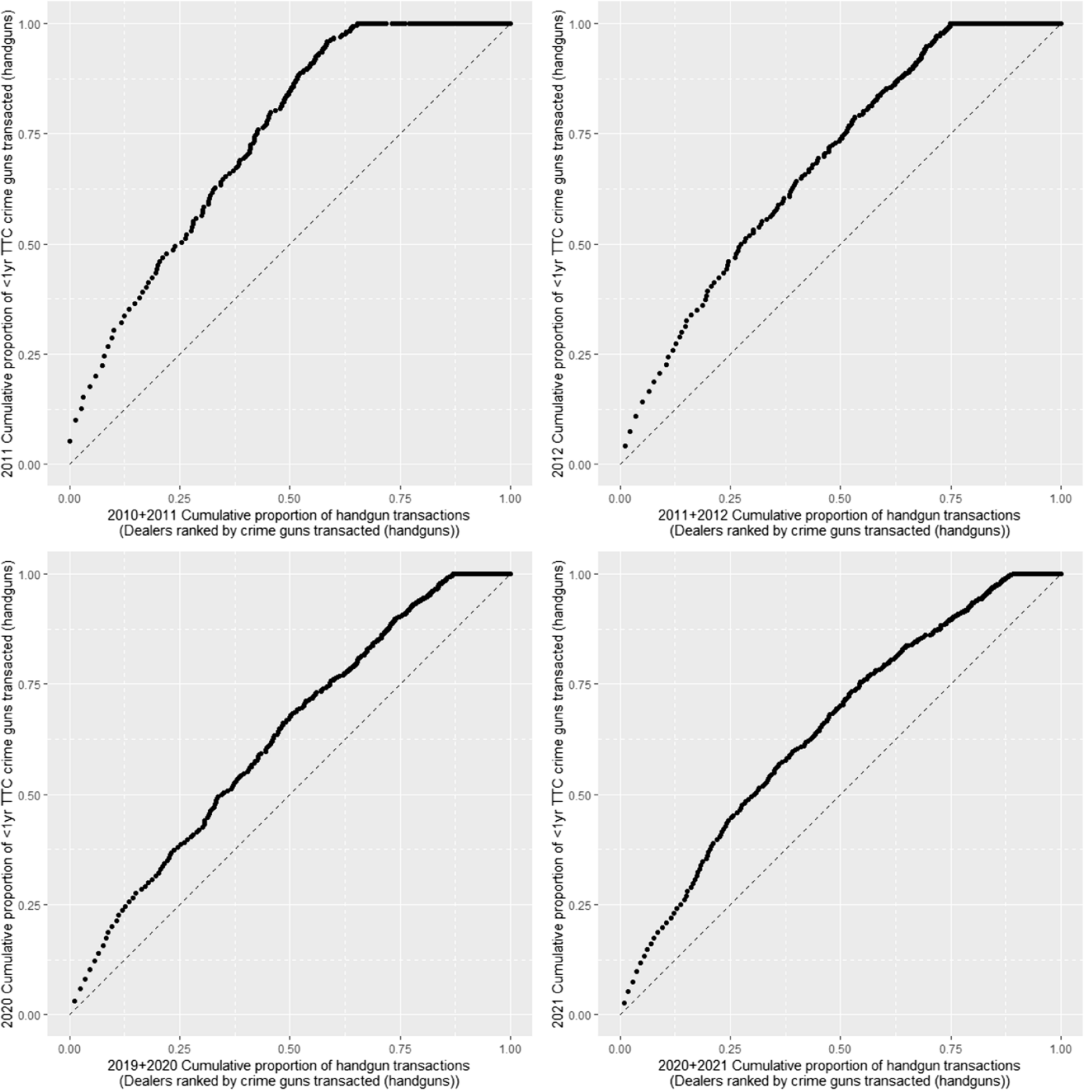
Trends in Dealer Distribution of Sales and Crime Guns: 2010–2012 and 2019–2021

## Retail Dealers as a Source of Crime Guns

Another potential source of crime guns are firearm dealers that either knowingly or through negligence supply firearms to traffickers, straw purchasers, or prohibited individuals. Survey and interview research has documented the willingness of some licensed dealers to provide criminals with firearms [29]. Studies using ATF trace data have also documented that a small fraction of retailers sell the vast majority of firearms later recovered by law enforcement [30]. An early and often cited finding from a 1998 ATF study reported that 1.2% of licensed dealers accounted for more than 57% of the crime guns [31]. However, these statistics do not account for retailer sales volume [32, 33]. Nonetheless, research has shown several retailer level risk-factors for crime gun sales, suggesting that the number of traced handguns sales is not *simply* a function of the number of total sales [32]. These predictors included dealer licensure as a pawnbroker and the percentage of sales that were denied following a background check [32].

In our data, without controlling for sales volume, we find 10% of FFLs account for 95% of crime guns; 15% of FFLs account for 98% of crime guns (Fig. 6, left panel). However, after scaling by dealer contribution to total sales volume (right panel), we find some disproportionate crime gun sales, but the concentration is much less stark. Adjusting for sales volume, approximately 12.5% of dealers account for close to 25% of crime guns. Among dealers with the most crime gun sales (top decile), an average of 2.4% (SD=2.9%) of sales are of firearms subsequently recovered in crime; among the bottom decile, none of their sales are crime guns.

Over the last 15–20 years, CADOJ has made efforts to shut down problematic dealers, and there is some evidence that there are fewer “dirty” dealers now than at the start of the study period. Figure 7 again shows the cumulative proportion of handgun transactions with dealers ranked from left to right by their contribution to crime gun sales, here focusing on crime guns recovered within 1 year of transaction. The first two panels present 2010–2011 sales and 2011–2012 recoveries; the third and fourth panel show sales in 2019–2020 and 2020–2021 recoveries. In 2010, 25% of retailers accounted for half of crime guns recovered within a year, and roughly 65% of dealers accounted for all crime guns recovered within a year. By 2019, 25% of dealers accounted for only 35% of crime guns recovered in the year, and more than 85% of dealers accounted for all crime guns recovered within a year. Sales in 2020 suggest a slight increase in the number and concentration of problematic retailers but still less than a decade prior.

Irrespective of the number of potentially problematic dealers, we generally find the same risk and protective factors among dealers that have been documented in prior research, supporting the hypothesis that there are some dealers that attract higher risk buyers [32]. Our data suggest dealers that have a larger share of denials among their total transactions are also more likely to sell firearms that are subsequently recovered in crime and, among crime guns, to sell firearms with a shorter TTC. Prospective firearm purchasers are denied purchase most often because of a previous prohibiting criminal offense; a larger share of denials is thus thought to reflect a larger purchaser base that is at increased risk of engaging in crime [32, 34]. Among the top decile of retailers with the highest percentage of denials out of total sales, an average of 24% of their transactions (SD=34%) are denials. A simple logistic regression of the dealer’s denial rate on the proportion of their sales that are crime guns indicates a statistically significant relationship (OR=1.14, 95% CI[1.13,1.15]). Likewise, a linear regression of dealer denial rate on TTC also suggests a statistically significant relationship: a 1% increase in the denial rate is associated with a 3.9 day reduction in mean TTC (95% CI[1.55,6.20]).

Our data are also largely consistent with previous research that has found pawnbrokers have higher rates of crime gun sales [32]. We examine the relationship between the proportion of a retailers’ transactions that are pawn transactions and crime gun recovery and TTC (among crime guns). Running a generalized linear model with three categories of retailers—those that have no pawn sales (approximately 61% of the approximately 1,380 FFLs active in a given year), those whose pawn sales volume is less than 50% of their total sales (approximately 37% of FFLs), and those whose firearm transactions consist of more than 50% pawn transactions (approximately 2.4%) of FFLs—we find dealers who do not engage in pawning are less likely to sell crime guns (OR=0.643, 95% CI[0.598,0.689]). However, we find that among the relatively few retailers for whom the bulk of their firearm transaction business is pawning, they are actually *less* likely to sell crime guns. This may be explained by generally low sales volume. Previous research has documented lower volume dealers to be linked to lower rates of crime gun recovery [9] and we find FFLs whose retail activity consists of more than half pawn transactions, sell, on average, fewer than 35 firearms a year and put up for pawn close to 700 per year. Those with some but less than 50% pawn transactions sell an average of 213,000 firearms per year (approximately 406 firearms per dealer). We find no statistically significant relationship between pawn sales and TTC.

## Discussion

Other than at the local level, there are few systematic data on crime-involved guns and their movement from on-the-books sales to criminal end user. This is the first study to analyze California’s statewide compilation of crime gun and stolen gun data. We document a significant rise in crime gun recoveries within a short period of time following legal purchase, which we see at both the state and local level. These trends correspond with rising legal handgun purchasing over the decade, supporting evidence that the prevalence of legal acquisition and ownership impacts the ease with which criminals can acquire firearms [24]. We also document the rise of PMFs across the state, though note substantial variation across cities in their prevalence. This variation may relate to differences in access to legal firearms across place. For example, the prevalence of PMFs is particularly high in San Francisco, a city in which the sale, distribution, and transfer of firearms and ammunition is prohibited. Notably, we also find that theft has been higher in San Francisco relative to many other cities, though declining over the last five years.

Overall, we find theft plays some role in directly arming criminals (6–8% of recovered crime guns had previously been reported stolen). The role of theft may be diminishing as individuals seeking firearms for illegal use may be less likely to resort to theft as PMFs have become more readily available. In 2022, California passed comprehensive legislation to regulate the ghost gun industry and ensure that the sale and manufacture of firearm precursor parts are subject to the same laws and regulations as fully assembled firearms [35]. It will be important to track the impact of these laws on the prevalence of PMFs recovered in crime.

We find evidence that some retailers contribute disproportionately to the supply of crime guns, though much less dramatically than statistics often cited would suggest. The data indicate that there may be somewhat fewer problematic dealers now than there were a decade ago. Conversations with CADOJ suggest that this may be the result of their efforts to shut down “dirty dealers.” A statewide dealer regulation was also passed in 2013, requiring all persons engaged in the business of selling firearms possess a state Certificate of Eligibility and be named on the state’s Centralized List of firearms retailers [36].

Even with fewer problematic dealers, the same risk factors persist: denials in particular are an indicator of a riskier retailer. Retailers’ denial percentage could provide a useful measure to prompt further investigation by law enforcement [32], and, insofar as this could aid efforts to disrupt the illegal supply of firearms, it highlights the utility of recording and maintaining the kind of detailed data on firearm transactions that is done in California. Data on sale denials is only possible in states that serve as the “point of contact” (POC)—i.e., states that have their own designated agencies to conduct background checks using state, as well as federal, records and databases. Currently, there are 13 POC states: California, Colorado, Connecticut, Florida, Hawaii, Illinois, Nevada, New Jersey, Oregon, Pennsylvania, Tennessee, Utah, and Virginia [37].

There are several limitations of this study to note. Even though reporting all recovered guns is mandatory under California state law, there is likely missingness in reporting. The number of annual crime gun reports are approximately 90% the number reported by ATF for the state of California, which suggests that most, but not all, gun trace requests received by ATF were submitted directly by law enforcement agencies through ATF’s E-trace portal to the CADOJ. However, this approximately 90% may not be consistent across jurisdictions. We know based on ATF trace data reported in recent research in Oakland, California [19], for example, that for this city we are missing a large fraction of ATF traces. Our further analyses of missingness (Supplement Section 1 suggests meaningful missingness from any single jurisdiction comprises less than 5% of total crime gun reports, and sensitivity analyses excluding these jurisdictions do not change any of our basic findings. Another limitation is that, given Tiahrt Amendment prohibitions, we do not have access to ATF trace results, so our records do not include source states for out-of-state purchases or out-of-state recoveries. This is necessarily a within-state study. While a limitation, the majority of crime guns recovered in California have an in-state sale, and we are able to estimate the fraction of out-of-state recoveries using aggregate ATF data [38]. Finally, as is always an issue with even complete ATF crime gun trace reports, trace requests may not represent all firearms recovered by law enforcement or be representative of firearms possessed and used by criminals [39]. Nonetheless, the general trends are still informative.

### Supplementary Information

Below is the link to the electronic supplementary material.

Supplementary file 1 (pdf 219 KB)
