# Supplementary material for: Trends and Sources of Crime Guns in California: 2010–2021
Source: J Urban Health. 2023 Sep 11;100(5):879–91. doi: 10.1007/s11524-023-00741-y (PMC10618133; doi:10.1007/s11524-023-00741-y)
Supplement: Supplementary file 1 — (pdf 219 KB) [file 11524_2023_741_MOESM1_ESM.pdf]

Supplementary Information

Trends and Sources of Crime Guns: 2010–2021

Hannah S. Laqueur, Colette Smirniotis, Christopher Mccort, Garen J. Wintemute

A Analysis of Potential Missingness

As noted in the manuscript, the California DOJ crime gun data represents roughly 90% of annual trace records reported by ATF for the state, depending on year. In all but a handful of jurisdictions, there does not seem to be any significant missingness across years. All medium and large California cities (those with populations of 200,000 or more, n = 22) reported in all study years. We further analyze missingness at the jurisdiction-level with the following process. First, we visually examined the crime gun reports for all law enforcement ORIs (Originating Agency Identifiers) that had: (1) at least 500 crime gun recoveries over the 11 year study period, (2) less than the annual average (40 crime gun reports) in at least one year, and (3) crime gun reports in fewer than 10 cities (so as to exclude statewide agencies such as highway patrol). Via visual inspection, we identified potentially problematic cities. We determined a city's trends to be reasonable and not likely containing significant missingness when the city agency crime gun report trends matched the aggregate trends of crime gun reports in the city provided by all other ORIs (law enforcement agencies may identify and report a crime gun in a city other than their reporting jurisdiction). We were left with 18 cities, of varying sizes, that appeared to have potential under-reporting during some years during the study period. Figure S.1 presents these cities with the red bars indicating the years that appear to be reliable and the blue bars indicating years that appear potentially suspect. As noted in the Discussion, we know based on ATF trace data that there is substantial missingness for the city of Oakland in the second half of our study period. This is reflected in the figure. To estimate the total missingness that might result from this under-reporting we assumed the average for each city during the the years when reporting appeared reliable. We then assumed this average for the years where the data appear potentially problematic. This amounted to approximately 4.5% of crime gun events (approximately 18,000 crime gun reports out of roughly 400,000 crime gun events.)

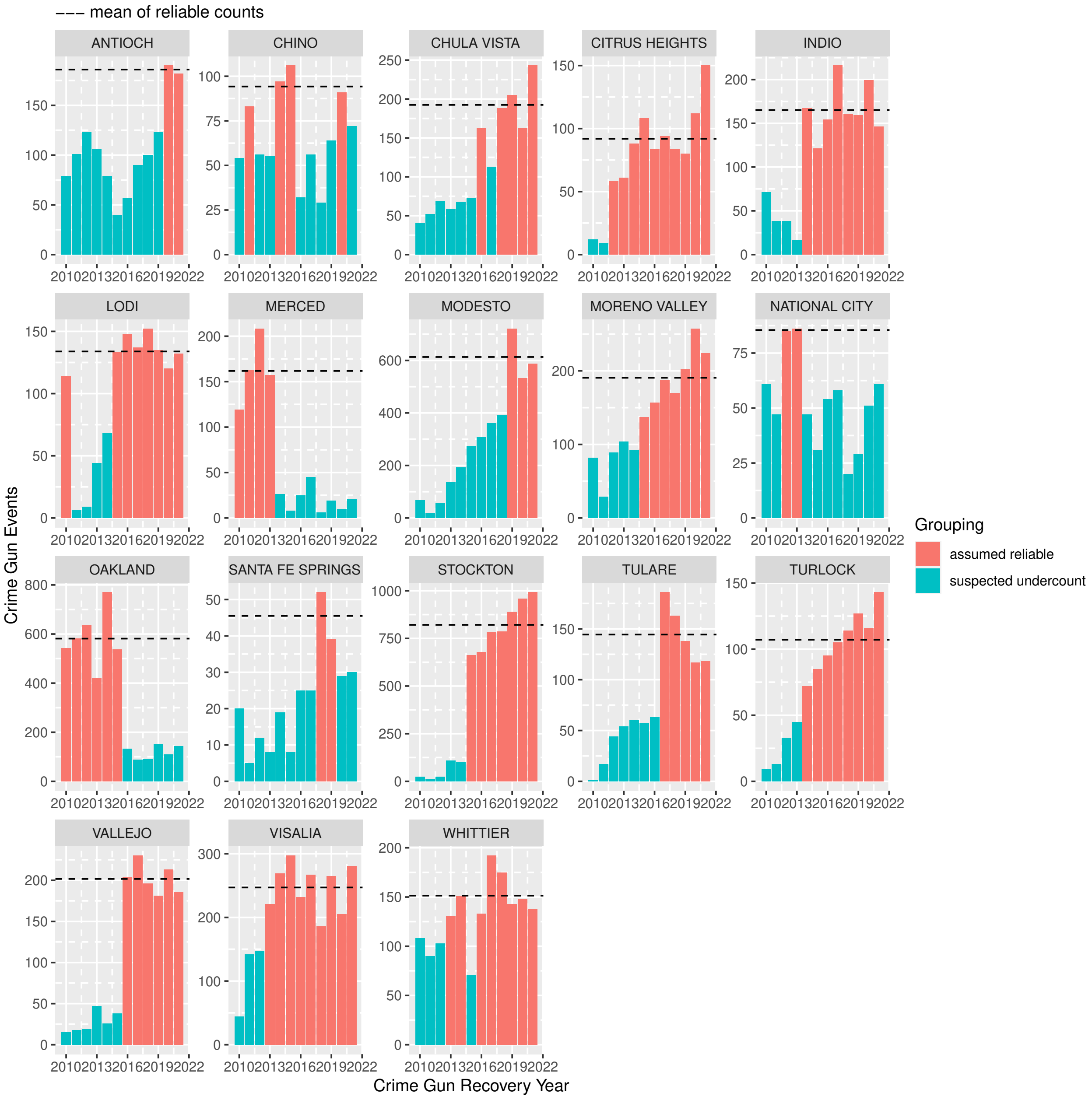

Figure S.1: Cities with Suspected Under-Reporting

B Trends in Crime Gun Recovery by Crime Type

Figure S.2 shows temporal trends in crime gun recovery by the crime type recorded by law enforcement. We present the most common offense categories, with a particular interest in violent crime. The panel on the right provides detail of the full plot (on the left) to better illustrate trends in the violent crime gun recoveries, which include homicide, robbery, assault, sexual violence, kidnapping and threats. Notably, over the study period, recoveries associated with assaults have nearly doubled; homicide recoveries have also increased. Recoveries associated with robberies have been relatively flat with a slight decline in the last two years.

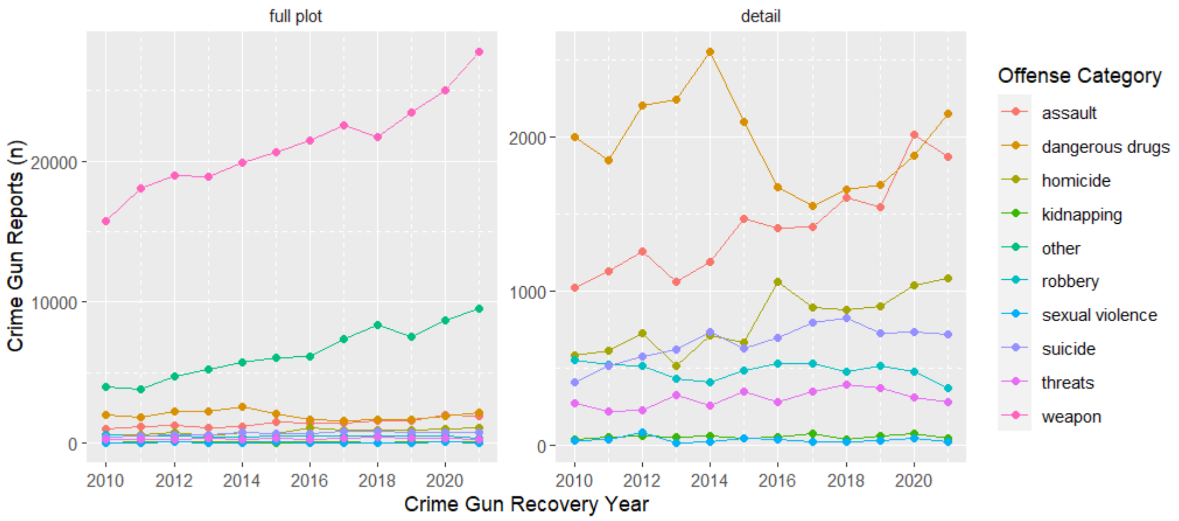

Figure S.2: Trends in Crime Gun Recovery by Crime Type

Table S.1 presents the CADOJ crime categories included in violent crime. We include the following crime types in violent crime: homicide, sexual assault, kidnapping, robbery, assault and threats.

UOC Chapter	UOC Category	Assigned Category	Assigned Crime Type
35	Dangerous Drug	Drugs	Drug
9	Homicide	Homicide	Violent
11	Sexual Assault	Sexual Violence	Violent
10	Kidnapping	Violence	Violent
12	Robbery	Violence	Violent
13	Assault	Violence	Violent
16	Threats	Violence	Violent

Table S.1: Uniform Offense Code Offense Categorization

C Legal Handgun Sales in California

Figure S.3 shows monthly handgun sales recorded in DROS. In 2010, monthly handgun sales averaged approximately 20,000 per month. There have been several spikes in handgun purchasing over the decade, for example, a significant increase in purchasing following the Sandy Hook School Shooting in December 2012 and the San Bernardino, California shooting in 2015. There was an even more dramatic spike at the outset of the COVID-19 pandemic in spring 2020, where monthly purchasing was close to 80,000.

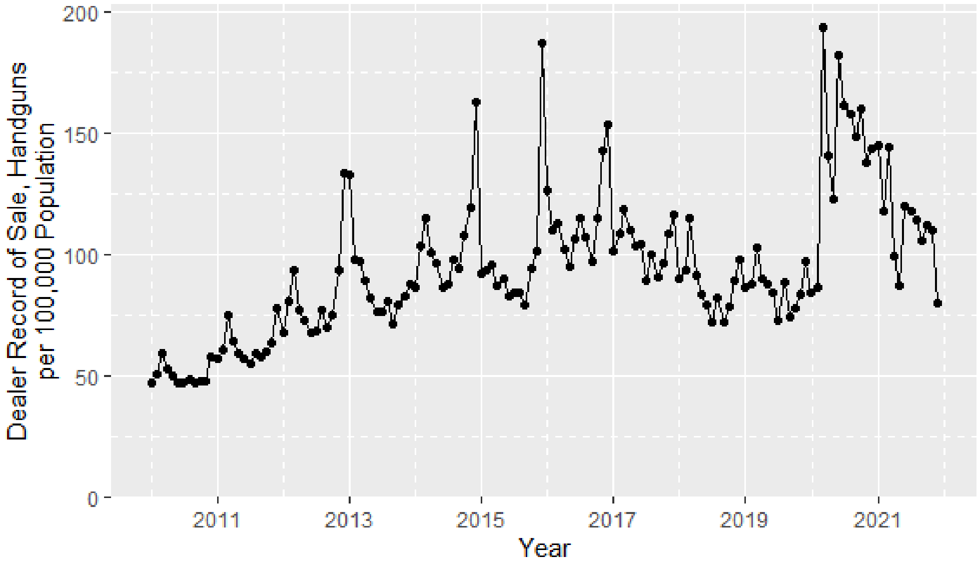

Figure S.3: Monthly Handgun Transactions: 2010–2021

D Trends in crime guns with in state and out-of-state records

Figure S.4 shows the fraction of recovered crime guns that had a previous record in California's Dealer Record of Sale (DROS) database. The fraction is consistently above 40% for most of the study period. In the last two years the fraction has dropped to below 30%. In part, these trends may represent the growth of PMF.

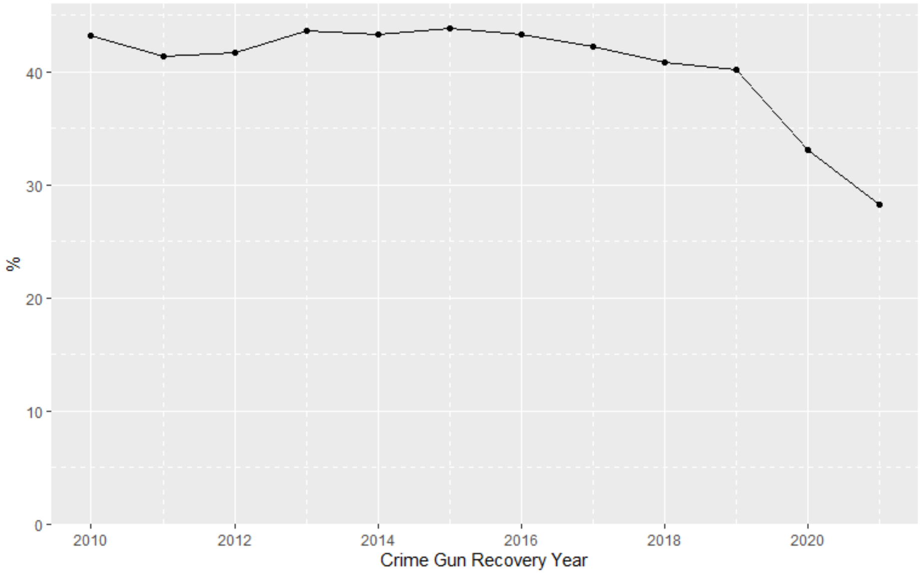

Figure S.4: Trends in Percentage of Handguns with a previous DROS record: 2010–2021

In addition to an increasing share of PMF crime guns, there is also data to suggest the fraction of crime guns originating from other states has been increasing. Annual ATF reports on firearm traces provide aggregate data that include the total number of firearms recovered and traced in the state in the calendar year, the number of recovered firearms that were successfully traced, and

the top 15 source states for those firearms (when a source state was identified). Figure S.5 shows the temporal trend in the share of traced crime guns that originated in states other than California (when the source state was identified).

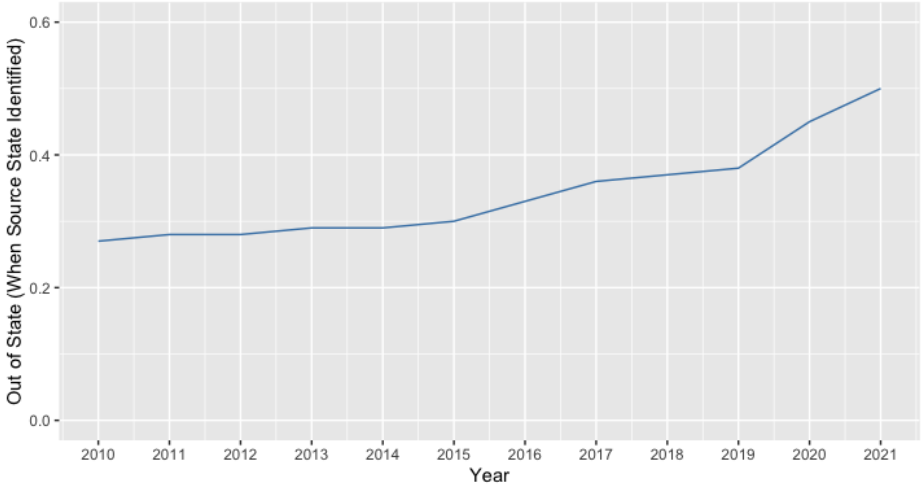

Figure S.5: Proportion of traced crime guns that originating out-of-state (when source state identified)

E Trends in Stolen Gun Recoveries

Figure S.6 shows the statewide monthly count of stolen gun reports. Table S.2 below presents the number of stolen gun reports each year, the percentage recovered within the year, and the percentage recovered by the end of the study period. If a stolen gun is recovered, the data suggest this occurs on average within less than a year, and most within three years. The cumulative recovery rate is only 15%.

Year Reported Stolen	# Stolen	% Recovered within 1 Year	% Cumulative (recovered by 2021)
2010	9692	6.7	14.3
2011	10923	6.4	13.7
2012	11920	7.3	14.6
2013	11696	7.7	15.0
2014	10545	7.3	14.8
2015	11509	8.6	15.0
2016	12041	8.9	15.3
2017	12018	9.4	15.1
2018	10591	9.9	14.5
2019	9880	9.2	12.2
2020	8932	10.1	11.3
2021	7906	6.7	6.7

Table S.2: Number of Stolen Gun Reports and Probability of Recovery

Figure S.7 presents the fraction of guns recovered in violent crime and other crimes that had been previously reported stolen.

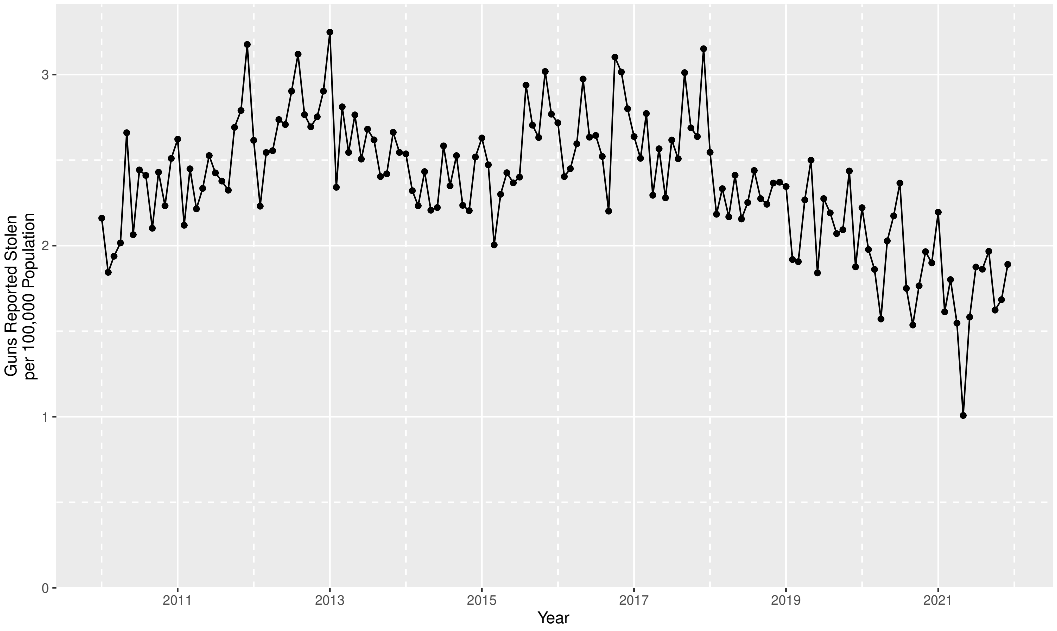

Figure S.6: Monthly Stolen Gun Reports: 2010–2021

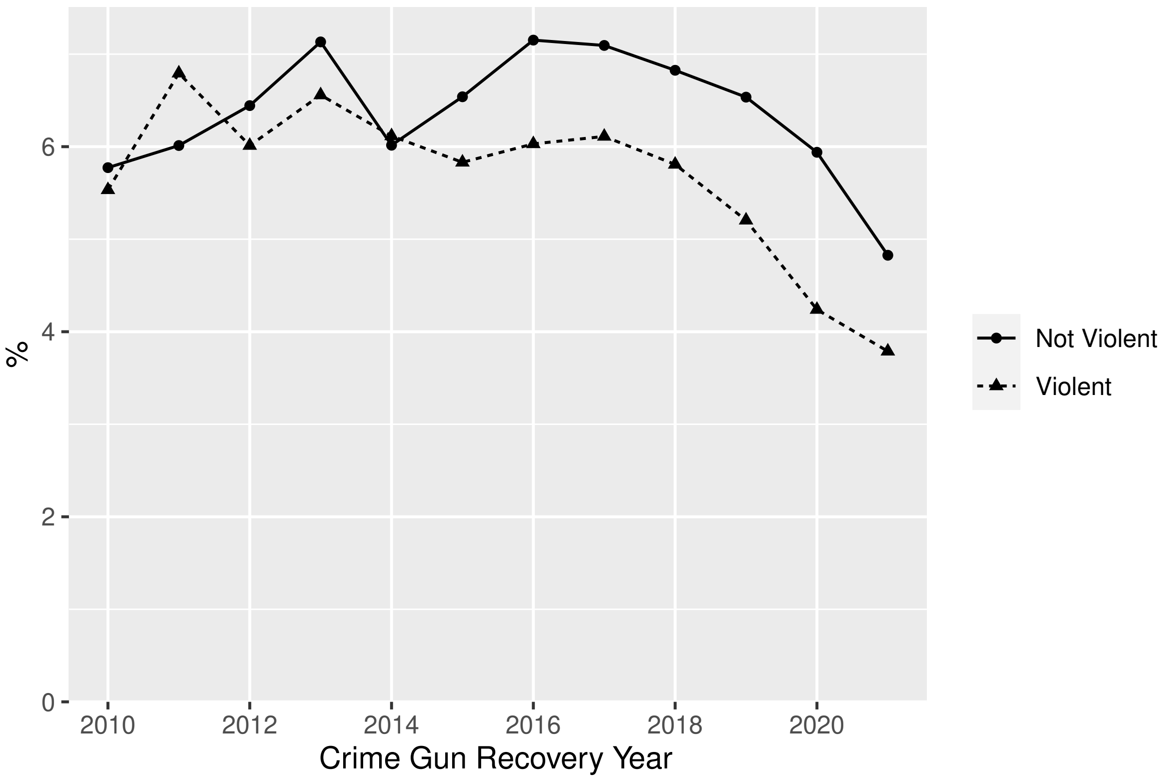

Figure S.7: Crime Guns Reported Stolen Prior to Recovery

Finally, figure S.8 presents the fraction of crime guns recovered across cities in the California that had previously been reported stolen. Figure S.9 presents a heat map showing the city in which a firearm was reported stolen and the city in which it was recovered. Lighter squares represent a higher probability of crime gun recovery. The figure illustrates there are some cities in which firearms appear to be stolen but then flow to other neighboring cities. In other cities, the location of the theft appears to be the same as the location of the law enforcement recovery.

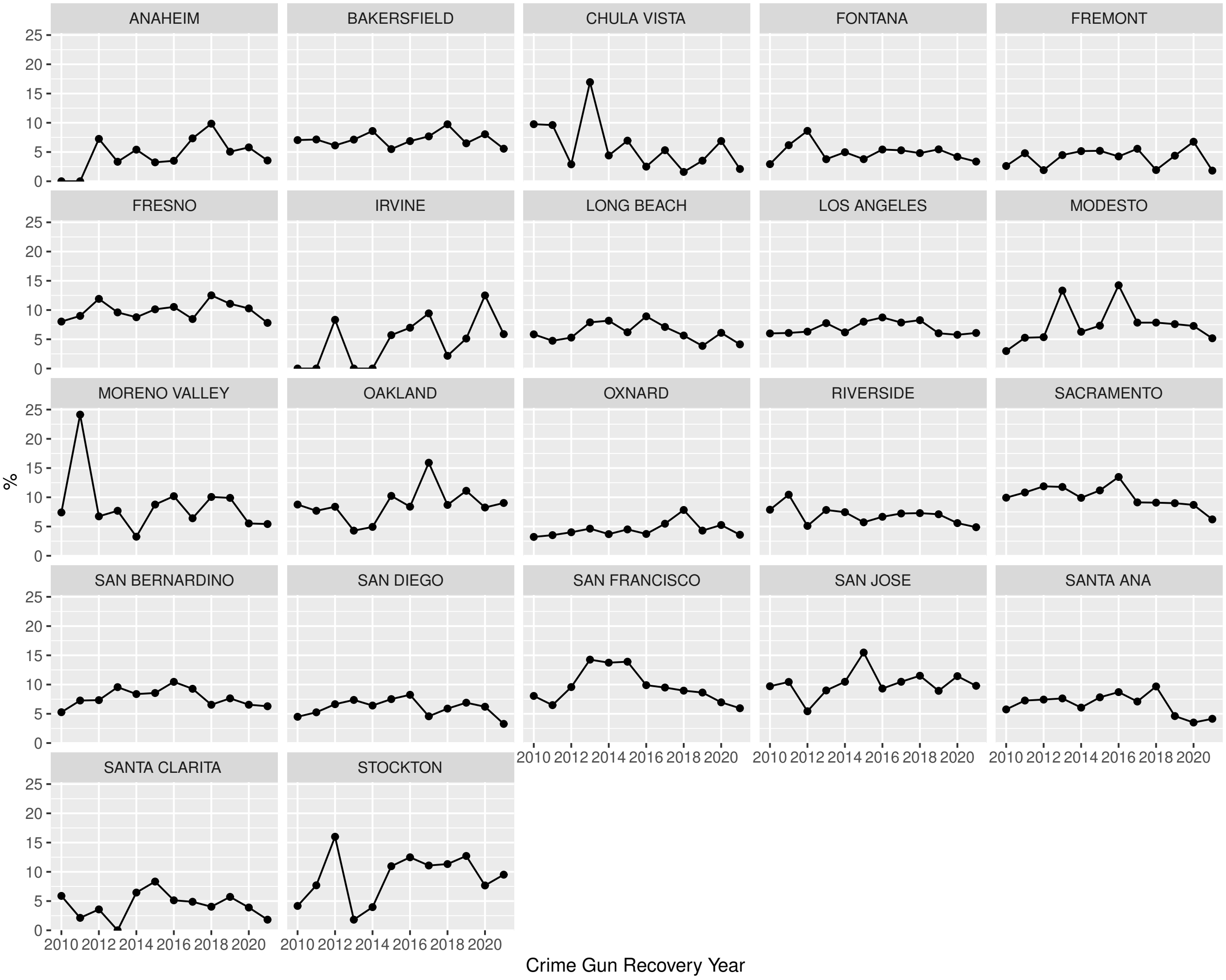

Figure S.8: Trends in the fraction of stolen crime gun by city (with populations 200,000 and above)

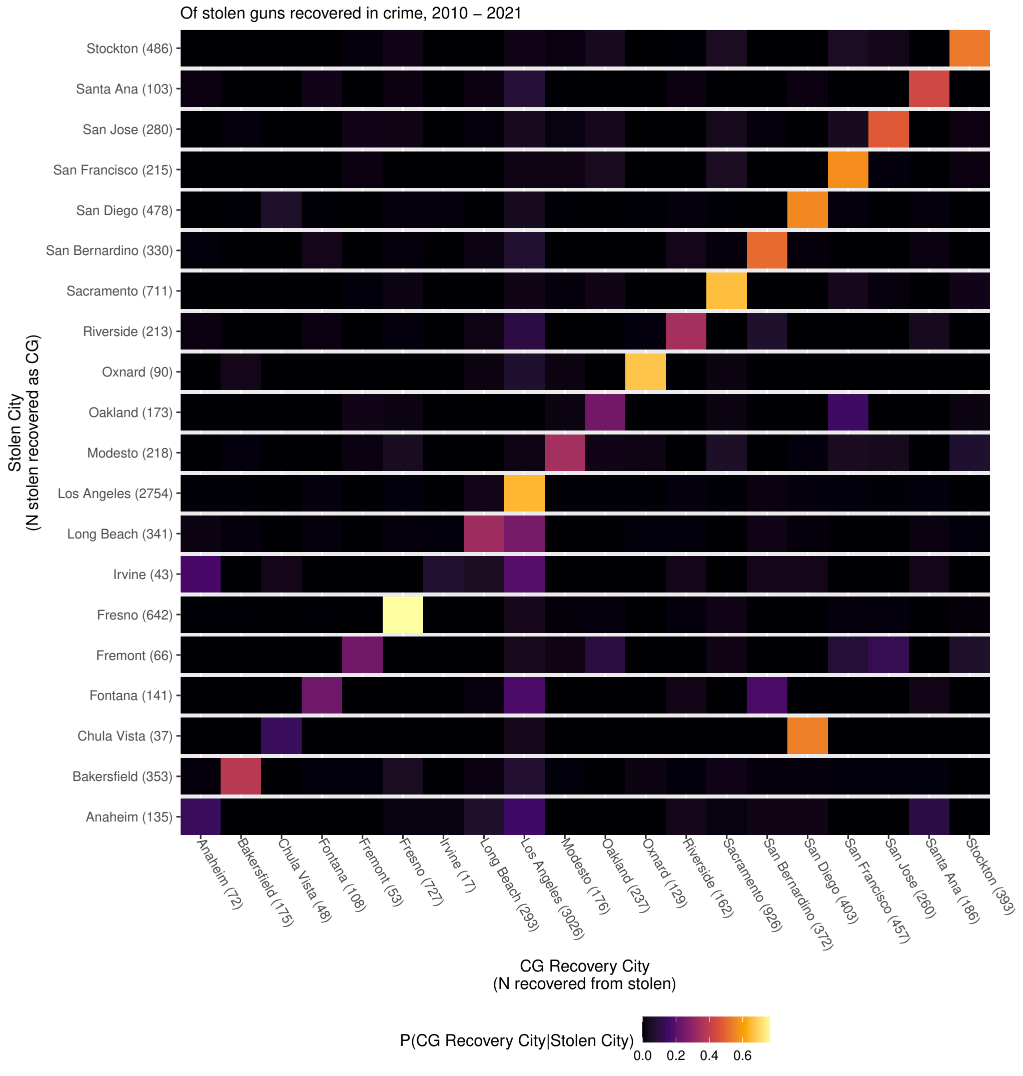

Figure S.9: Heat Map of City of Stolen Gun Report vs City of Crime Gun Recovery
